# Myeloid dendritic cells display downregulation of C-type lectin receptors and aberrant lectin uptake in systemic lupus erythematosus

**DOI:** 10.1186/ar2517

**Published:** 2008-09-23

**Authors:** Seetha U Monrad, Kristine Rea, Seth Thacker, Mariana J Kaplan

**Affiliations:** 1 Division of Rheumatology, Department of Internal Medicine, University of Michigan Medical School, 1150 West Medical Center Drive, 5520 MSRBI, Ann Arbor, MI 48109, USA

## Abstract

**Introduction:**

There is a growing body of evidence implicating aberrant dendritic cell function as a crucial component in the immunopathogenesis of systemic lupus erythematosus. The purpose of the present study was to characterize the phagocytic capacity and expression of receptors involved in pathogen recognition and self-nonself discrimination on myeloid dendritic cells from patients with lupus.

**Methods:**

Unstimulated or stimulated monocyte-derived dendritic cells were obtained from lupus patients and healthy control individuals, and expression of C-type lectin receptors (mannose receptor and dendritic cell-specific intercellular adhesion molecule-grabbing nonintegrin), complement-receptor 3 and Fcγ receptors was determined by flow cytometry. Dextran uptake by lupus and control dendritic cells was also assessed by flow cytometry. Serum IFNγ was quantified by ELISA, and uptake of microbial products was measured using fluorescently labeled zymosan.

**Results:**

When compared with dendritic cells from healthy control individuals, unstimulated and stimulated lupus dendritic cells displayed significantly decreased dextran uptake and mannose receptor and dendritic cell-specific intercellular adhesion molecule-grabbing nonintegrin expression. Decreased expression of the mannose receptor was associated with high serum IFNγ levels, but not with maturation status or medications. Diminished dextran uptake and mannose receptor expression correlated with lupus disease activity. There were no differences between control and lupus dendritic cells in the expression of other pattern recognition receptors or in the capacity to uptake zymosan particles

**Conclusions:**

Lupus dendritic cells have diminished endocytic capacity, which correlates with decreased mannose receptor expression. While this phenomenon appears primarily intrinsic to dendritic cells, modulation by serum factors such as IFNγ could play a role. These abnormalities may be relevant to the aberrant immune homeostasis and the increased susceptibility to infections described in lupus.

## Introduction

Systemic lupus erythematosus (SLE) is an autoimmune disease with protean clinical manifestations, typically characterized by the presence of autoantibodies to nuclear components and by the deposition of immune complexes in various tissues. While many cell types have been implicated as pathogenic in this disease, a growing body of literature demonstrates the potential role that dendritic cells (DCs) may play in the development and perpetuation of disease in SLE (reviewed in [1]). DCs regulate both innate and adaptive immune effector cells, and have powerful and widespread effects on all aspects of the immune system. Breakdown of DC regulation can lead to loss of tolerance at multiple levels, and can thereby promote autoimmune responses. Additionally, plasmacytoid DCs are the primary cellular producers of type I interferons – cytokines strongly implicated in SLE immunopathogenesis [2].

Myeloid DCs reside in an inactive, highly phagocytic state at sites of potential antigen exposure. Uptake of harmless environmental or self-antigens (often products of normal cellular senescence, apoptosis or necrosis) results in low-level migration to regional lymph nodes, where antigen presentation induces tolerance or anergy in resident lymphocytes. Uptake of pathogenic antigens in the presence of other stimulatory signals induces DC maturation, manifested by downregulation of phagocytic receptors and upregulation of antigen-presentation machinery, and migration to lymphoid tissues to trigger secondary specific immune responses. DCs are therefore crucial for generating and maintaining peripheral tolerance, a key component in the prevention of autoimmunity, as well as stimulating immune responses in appropriate settings [3].

Abnormal DC function could result in aberrant uptake and presentation of harmless self-antigen, triggering inappropriate immune responses to self, a hallmark of SLE. It could also lead to inadequate response to truly pathogenic stimuli, with resultant inability to properly combat infections. This also is of potential relevance in lupus, as individuals with this disease have significant morbidity/mortality from infections. Whether the poor outcomes after infection are secondary to intrinsic abnormalities in immune function seen in this disease or to the use of immunosuppressive medications, however, is unclear [4,5].

A crucial aspect of normal DC function is to discriminate between harmless self-antigens and potentially harmful foreign antigens. To this end, DCs express a number of pattern recognition receptors, which recognize specific molecular patterns exhibited on a variety of cell types and pathogens. Among these are the C-type lectin receptors (CTLRs). The CTLRs comprise a family of evolutionarily conserved proteins containing one or more C-type lectin domains, and may bind carbohydrate moieties in a calcium-dependent manner [6]. CTLRs can recognize pathogen-associated molecular patterns expressed on microbes, as well as ligands expressed on apoptotic and malignant endogenous cells. Additionally, they can interact with other pattern recognition receptors such as Toll-like receptors. DCs express a number of different membrane-bound CTLRs, which can function as pathogenic antigen-recognition and antigen-uptake receptors, internalizing and processing for efficient presentation to effector cells. CTLRs can also recognize endogenous glycoproteins and can bind cellular adhesion molecules, thus having roles in homeostatic clearance and migration (reviewed in [7-9]).

One DC-associated CTLR is the mannose receptor (MR), CD206. This type I transmembrane protein is expressed by both macrophages and DCs, and has numerous ligands including bacterial cell wall components [10] and endogenous glycoproteins (lysosomal hydrolases) [11]. The MR internalizes antigens to early endosomes before recycling back to the surface. Antigens are subsequently processed for presentation on Major Histocompatibility Complex (MHC) molecules as well as (in the case of the *Mycobacterium tuberculosis *lipoarabinomannan component [12]) on CD1b.

Another CTLR expressed exclusively by human myeloid DCs is the DC-specific intercellular adhesion molecule-grabbing nonintegrin (DC-SIGN), CD209. A type II transmembrane protein, DC-SIGN binds intercellular adhesion molecule 2 (on endothelial cells) and intercellular adhesion molecule 3 (on leukocytes), thereby regulating DC migration and T-cell interactions [13,14]. DC-SIGN also is involved in the transport of HIV-1 for subsequent transinfection of CD4^+ ^T cells [15]. Other DC-associated CTRLs include DEC-205 (CD205) and DC-associated C-type lectin-1 (Dectin-1), an important binder of β-glucan.

DCs express other uptake receptors involved in pathogen recognition and self-nonself discrimination [16]. Type III complement receptor (CR3), CD11b/CD18, is a β_2_-integrin that serves both as an adhesion molecule and a myeloid phagocytic receptor for complement-opsonized particles [17]. Fcγ receptor I (CD64), Fcγ receptor II (CD32) and Fcγ receptor III (CD16) are present on different subsets of human DCs. In addition to binding immunoglobulin-opsonized particles, ligation of Fcγ receptor II by nucleic acid-containing immune complexes can trigger IFNα production by plasmacytoid DCs [18,19]. Recent genome-wide association studies in lupus patients have identified single nucleotide polymorphisms in or near *ITGAM *and *FCGR2A *(the genes for CR3 and Fcγ receptor II, respectively) [20], supporting a potential role for variants of these genes in lupus susceptibility.

Our group has previously demonstrated that monocyte-derived DCs (moDCs) from human SLE patients display an activated phenotype, characterized by accelerated differentiation, increased baseline maturation, augmented synthesis of proinflammatory cytokines, and increased ability to promote increased proliferation and activation of allogeneic control T cells [21]. In the present study, we investigated the endocytic capacity and surface expression of different pattern recognition receptors in SLE moDCs.

## Materials and methods

### Patient selection

The study was approved by the University of Michigan Medical Institutional Review Board and the research was in compliance with the Helsinki Declaration. Written informed consent was obtained for all patients.

Patients fulfilling the American College of Rheumatology criteria for SLE [22,23] were recruited during routine outpatient rheumatology clinic visits as well as during inpatient admissions at the University of Michigan. Patients were excluded if they had undergone or were undergoing treatment for concurrent malignancy or they had significant clinical overlap with another autoimmune condition. Healthy control individuals were obtained by advertisement.

The SLE activity was assessed by the SLE Disease Activity Index [24]. Patient cells and control cells were cultured and analyzed in parallel. Information regarding the demographics, disease activity, and use of medications is presented in Table 1. Only two patients had evidence of active lupus nephritis and one patient had active lupus cerebritis. The majority of SLE clinical manifestations were cutaneous, arthritic or hematologic.

**Table 1:** Demographic and clinical characteristics of systemic lupus erythematosus patients

Characteristic	Systemic lupus erythematosus patients	Control individuals
Number	63	31
Female (%)	85.7	67.8
Age, mean (range) (years)	40.6 (21 to 67)	31.9 (23 to 54)
Systemic Lupus Erythematosus Disease Activity Index (mean)	4.2 ± 0.4	
Systemic Lupus Erythematosus Disease Activity Index >2 (%)	57.2	
Elevated dsDNA antibodies (%)	58.7	
Decreased C3 and/or C4 (%)	31.7	
Medications (%)		
Antimalarials	73.0	
Azathioprine	4.8	
Cyclophosphamide	4.8	
Methotrexate	4.8	
Mycophenolate	30.2	
Prednisone (%)		
None	36.5	
<30 mg/day	52.4	
>30 mg/day	11.1	
No medications (%)	14	

### Reagents

Human recombinant IL-4, TNFα, and IFNγ were purchased from PeproTech (Rocky Hill, NJ, USA). Human granulocyte-macrophage colony-stimulating factor was either purchased (recombinant) from Invitrogen (Carlsbad, CA, USA) or kindly donated from Berlex (Montville, NJ, USA).

The culture media for DCs included X-vivo 15 serum-free media (BioWhittaker, Walkersville, MD, USA), RPMI1640, fetal calf serum, L-glutamine and penicillin/streptomycin/amphotericin B (Gibco/Invitrogen, Carlsbad, CA, USA). Lipopolysaccharide (O26:B6), D-mannose, and FITC-dextran (FD; 40 kDa) were purchased from Sigma (St Louis, MO, USA).

Anti-human mAbs and their appropriate isotype controls conjugated to FITC, Phycoerythrin, allophycocyanin, and CyChrome were purchased from BD Biosciences (San Jose, CA, USA), from Ancell (Bayport, MN, USA), and from Biolegend (San Diego, CA, USA). These mAbs include anti-CD11c, CD11b, CD14, CD16, CD32, CD64, CD209, CD206, CD40, CD80, CD83, CD86, and class 2. Unlabeled zymosan A and zymosan A fluorescent BioParticles were purchased from Molecular Probes/Invitrogen (Carlsbad, CA, USA).

### Generation and stimulation of monocyte-derived dendritic cells

The moDCs were obtained as previously described [21]. Human peripheral blood mononuclear cells were isolated from whole blood by standard density gradient centrifugation on Ficoll-Hypaque Plus (Amersham Biosciences, Sweden) and were resuspended at 6 × 10^6 ^cells/ml in RPMI 1640 with antibiotics, L-glutamine and 10% fetal bovine serum. Cells were transferred to tissue culture plates, and monocytes were allowed to adhere to the plastic surface for 1 hour at 37°C. Nonadherent cells were removed by washing with PBS, and monocytes were further cultured for 5 days in DC growth medium (serum-free X-vivo-15 containing antibiotics, 50 ng/ml granulocyte-macrophage colony-stimulating factor and 5 ng/ml IL-4). At days 5 to 7, cells were harvested for immediate analysis, or stimulated with 1 μg/ml LPS and 100 ng/ml TNFα for an additional 48 hours prior to harvest.

### FITC-dextran uptake

Harvested moDCs were washed and resuspended in RPMI/antibiotics/10% fetal bovine serum with or without D-mannose (100 mg/ml). Cells were preincubated for 15 minutes at either 4°C or 37°C, followed by incubation for 1 hour with FD (1 mg/ml). The uptake reaction was terminated by washing three times with ice-cold PBS, followed by staining as described below.

### Zymosan uptake

Fluorescently labeled and unlabeled zymosan was reconstituted in 2 mM sodium azide/PBS (Sigma, St. Louis, MO, USA) as per the manufacturer's protocol to a concentration of 20 mg/ml each. As preliminary experiments revealed that fluorescein-labeled zymosan particles saturated the FITC channel of the flow cytometer and were not fully quenchable by acidic trypan blue, fluorescein zymosan was diluted 1:100 in unlabeled zymosan. Immature moDCs were harvested and preincubated as above, followed by addition of the diluted zymosan mixture to achieve a ratio of 1 DC:10 particles zymosan (25 μl zymosan mixture per 1 million DCs). Incubation, washing, processing, and flow cytometry was performed as for the FD experiments.

### Immunofluorescence staining and flow cytometry

DCs were washed with PBS/0.2% BSA, and Fc receptors were blocked by incubating cells for 20 minutes with 50% control human plasma. DCs were then incubated for 30 minutes at 4°C with 0.06 to 0.15 μg/ml fluorochrome-conjugated mAbs or appropriate isotype-matched control antibodies following the manufacturer's directions. Cells were then washed three times with PBS/0.2% BSA, fixed in 2% w/v paraformaldehyde, and analyzed on the FACSCalibur (BD Biosciences) and EPICS XL flow cytometers (Beckman Coulter, Fullerton, CA).

Data analysis was performed using WinMDI 2.8 software. Stained cells were gated by side-scatter and forward-scatter characteristics and were further identified by surface markers. The results were expressed as the percentage of cells staining positive for different markers as well as by mean channel fluorescence. The cutoff point for positive staining was above the level of the control isotype antibodies.

### IFNγ measurement

Plasma was collected from patient samples during peripheral blood mononuclear cell isolation and frozen at -80°C until use. IFNγ plasma levels were determined by ELISA using Ready-Set-Go kits with precoated plates (eBioscience, San Diego, CA, USA) as per the manufacturer's protocol.

### Drug treatment

Monocytes were cultured to induce DC differentiation in the presence or absence of graded concentrations of indomethacin (0.01 to 1 μg/ml), hydroxychloroquine (0.02 to 2 μg/ml), hydrocortisone (0.01 to 1 μM), 6-mercaptopurine (0.01 to 1 μM) and mycophenolate-mofetil (0.04 to 4 μg/ml) (all obtained from Sigma-Aldrich) or vehicle, as described previously [21].

### Statistical analysis

Data are expressed as the mean ± standard error of the mean. *P *values were calculated using two-tailed Student's *t*-tests. All correlations were calculated using the Spearman's rank correlation test.

## Results

### SLE dendritic cells exhibit diminished FITC-dextran uptake

**Figure 1:**
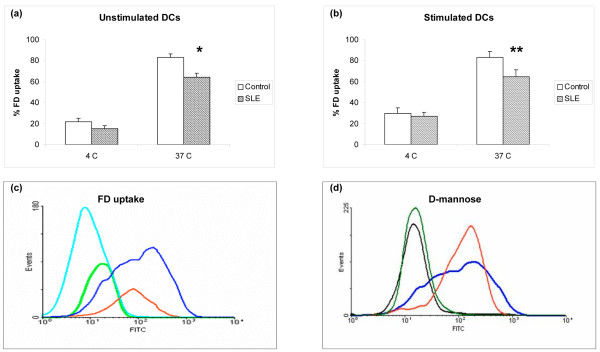
**Lupus monocyte-derived dendritic cells display decreased FITC-dextran uptake**. **(a) **Unstimulated dendritic cells (DCs) (*P = 0.003). **(b) **Lipopolysaccharide/TNFα-stimulated DCs (**P = 0.05). Results are expressed as the mean ± standard error of the mean. **(c) **Representative histogram demonstrating impaired FITC-dextran (FD) uptake by unstimulated systemic lupus erythematosus (SLE) DCs. **(d) **Representative histogram showing blunted FD uptake by unstimulated DCs after D-mannose preincubation. Line colors: dark blue = control, 37°C; red = SLE, 37°C; light blue = control, 4°C; light green = SLE, 4°C; black = control + D-mannose, 37°C; dark green = SLE + D-mannose, 37°C.

We first demonstrated that moDCs from SLE patients have impaired endocytic capacity. Healthy control moDCs exhibited low basal FD uptake at 4°C, which significantly increased when cells were incubated at 37°C. Lupus moDCs, however, exhibited decreased uptake of FD, both before (percentage uptake: control (*n *= 20), 83.1 ± 3.2 versus SLE (*n *= 47), 63.9 ± 3.9; *P *= 0.003; Figure 1a,c) and after stimulation with LPS and TNFα (percentage uptake: control (*n *= 13), 83.1 ± 5.8 and SLE (*n *= 30), 64.6 ± 6.5; *P *= 0.05; Figure 1b). FD uptake was blunted by preincubation of cells with D-mannose (Figure 1d), confirming that a mannose-dependent uptake mechanism is involved.

### SLE dendritic cells have decreased surface mannose receptor expression, which correlates with FITC-dextran uptake

**Figure 2:**
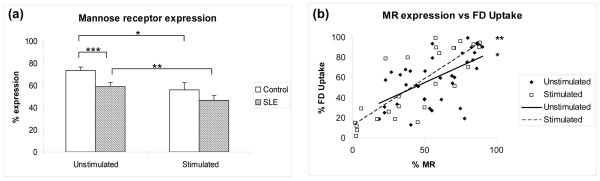
**Unstimulated lupus monocyte-derived dendritic cells, mannose receptor expression, and FITC-dextran uptake**. Unstimulated lupus monocyte-derived dendritic cells display decreased mannose receptor (MR) expression, which correlates with FITC-dextran (FD) uptake. **(a) **Both groups significantly downregulate MR upon lipopolysaccharide/TNF stimulation (*P = 0.007, **P = 0.03, ***P = 0.0002). Results are expressed as the mean ± standard error of the mean. **(b) **Positive correlation between MR expression and FD uptake. This was observed in unstimulated lupus DCs (*r = 0.64, *P *< 0.0001) and stimulated lupus DCs (**r = 0.80, *P *< 0.0001).

As the MR is the major receptor responsible for FD uptake inhibited by mannose, we then assessed MR expression in SLE moDCs and in control moDCs (Figure 2a). Both lupus and control moDCs expressed surface MR, which significantly downregulated upon stimulation/maturation with LPS and TNFα (*P *= 0.03 for lupus DCs, *P *= 0.007 for control). Immature moDCs from lupus patients, however, displayed significantly less MR when compared with control moDCs (percentage expression: control (*n *= 29), 73.6 ± 2.7 and SLE (*n *= 49), 59.2 ± 3.5; *P *= 0.0002). This difference was not significant after DC stimulation. Linking levels of MR to C-type lectin uptake, there was a positive correlation between MR expression and FD uptake in both unstimulated (*r *= 0.64) and stimulated (*r *= 0.80) lupus moDCs (*P *< 0.0001; Figure 2b).

### Decreased mannose receptor expression correlates with circulating IFNγ

**Figure 3:**
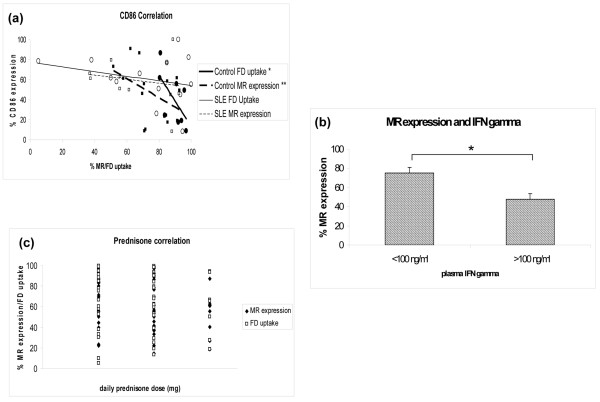
**Mannose receptor expression in systemic lupus erythematosus**. Mannose receptor (MR) expression is not correlated with CD86 expression or prednisone use, but is associated with high serum IFNγ in systemic lupus erythematosus (SLE) patients. **(a) **Correlation between CD86 expression and either FITC-dextran (FD) uptake or MR expression. In control dendritic cells (DCs) there is significant or near-significant negative correlation (*FD uptake: *r *= -0.76, *P *= 0.03; **MR expression: *r *= -0.46, *P *= 0.08), whereas there is no correlation in lupus DCs (FD uptake: *r *= -0.23, *P *= 0.46; MR expression: *r *= -0.33, *P *= 0.33). **(b) **Patients with higher levels of circulating IFNγ display lower expression of MR on autologous DCs. Graph displays patients with IFNγ levels >100 ng/ml (*n *= 3) compared with patients with lower plasma levels (*n *= 12; *P = 0.03). Results are expressed as the mean ± standard error of the mean. **(c) **Prednisone dose does not correlate with MR expression or FD uptake. Graph shows the distribution of MR expression (black diamonds) and FD uptake (clear squares) relative to the prednisone dose.

We next investigated potential factors contributing to the observed aberrant phenotype in DCs from SLE patients. To determine whether the DC maturation status could account for the diminished FD uptake and MR expression, the association with expression of the maturation marker CD86 was examined (Figure 3a). Whereas in unstimulated control DCs there was a significant negative correlation between CD86 expression and FD uptake (*r *= -0.76, *P *= 0.03) and there was a near-significant negative correlation with MR expression (*r *= -0.46, *P *= 0.08), this was not found in unstimulated lupus DCs (*r *= -0.23, *P *= 0.33 for FD uptake; *r *= -0.33, *P *= 0.46 for MR expression). Similarly, no correlation was found with other maturation markers, including CD40, CD80, CD83 and class II Major Histocompatibility Complex (data not shown).

We also examined whether medications commonly used to treat lupus could account for decreased FD uptake or MR expression in this disease. There was no correlation of these variables with the prednisone dosage (Figure 3c) or with the use of nonsteroidal anti-inflammatory drugs, hydroxychloroquine, azathioprine, or mycophenolate (data not shown). Additionally, healthy control moDCs cultured in the presence of graded doses of the above medications did not exhibit decreased FD uptake or MR expression when compared with autologous untreated DCs (data not shown).

Overall, these results indicate that the abnormal phenotype and function of this CTLR are not secondary to a drug factor or DC maturation status.

IFNγ downregulates transcription and surface expression of the MR, and elevated levels of this cytokine have been described in SLE [25]. To assess whether CTLR abnormalities were secondary, at least in part, to this cytokine, plasma levels of IFNγ were quantified. Indeed, SLE individuals with IFNγ concentration >100 ng/ml had significantly lower moDC MR expression than those with lower levels (percentage expression: <100 ng/ml (*n *= 12), 75.2 ± 5.4 and >100 ng/ml (*n *= 3), 48.1 ± 5.6; *P *= 0.02; Figure 3b).

### Decreased mannose receptor expression and endocytic capacity correlates with lupus disease activity

**Figure 4:**
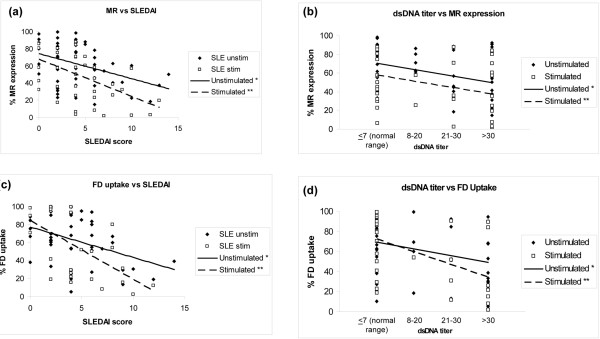
**Mannose receptor expression correlation with disease activity in monocyte-derived dendritic cells**. Disease activity and levels of anti-dsDNA antibody correlate with lower levels of mannose receptor (MR) and aberrant dextran uptake by lupus monocyte-derived dendritic cells. **(a) **Correlation between MR expression and systemic lupus erythematosus Disease Activity Index (SLEDAI) scores (*r = -0.36, *P *= 0.006; **r = -0.48, *P *= 0.002) in systemic lupus erythematosus (SLE) patients. **(b) **Correlation between anti-dsDNA antibodies and MR expression (*r = -0.35, *P *= 0.01; **r = -0.33, *P *= 0.04). **(c) **Correlation between lupus dendritic cell FITC-dextran (FD) uptake and SLEDAI scores (*r = -0. 34, *P *= 0.02; **r = -0.55, *P *= 0.001). **(d) **Correlation between anti-dsDNA antibody titers and FD uptake (*r = -0.29, *P *= 0.05; **r = -0.49, *P *= 0.004).

In both unstimulated and stimulated lupus moDCs, the MR expression negatively correlated with SLE Disease Activity Index scores (unstimulated, *r *= -0.36, *P *= 0.006; stimulated, *r *= -0.48, *P *= 0.002; Figure 4a) and with serum anti-dsDNA titers (unstimulated, *r *= -0.35, *P *= 0.01; stimulated, *r *= -0.33, *P *= 0.04; Figure 4b). Similar negative correlations were observed between the FD uptake and SLE Disease Activity Index scores (unstimulated, *r *= -0. 34, *P *= 0.02; stimulated, *r *= -0.55, *P *= 0.001; Figure 4c) or anti-dsDNA (unstimulated, *r *= -0.29, *P *= 0.05; stimulated, *r *= -0.49, *P *= 0.004; Figure 4d).

### Lupus dendritic cells display decreased DC-SIGN, but present normal CR3 and Fcγ receptor expression

To determine whether this endocytic defect was restricted to the MR or whether other receptors were also aberrantly expressed, the surface expression of other receptors involved in antigen uptake was evaluated. There were no differences in CR3 and Fcγ receptor I, Fcγ receptor II or Fcγ receptor III expression between lupus DCs and control DCs (data not shown).

**Figure 5:**
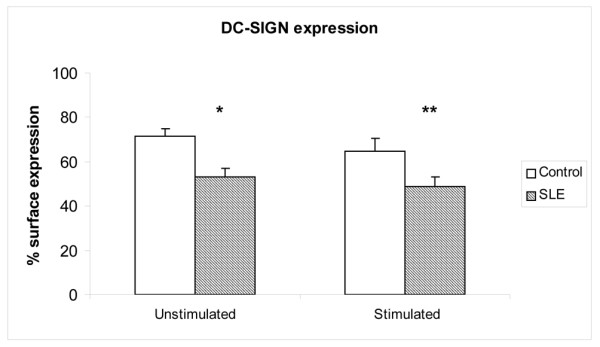
**Dendritic cell-specific intercellular adhesion molecule-grabbing nonintegrin expression in systemic lupus erythematosus dendritic cells**. Dendritic cell-specific intercellular adhesion molecule-grabbing nonintegrin (DC-SIGN) expression is downregulated in unstimulated and stimulated systemic lupus erythematosus (SLE) monocyte-derived dendritic cells. Results represent the mean ± standard error of the mean of 30 control individuals and 52 SLE patients (*P = 0.005, **P = 0.03).

The CTLR DC-SIGN, however, was also downregulated in SLE moDCs – both before and after stimulation (unstimulated percentage expression: control (*n *= 30), 71.3 ± 3.5 and SLE (*n *= 52), 53.2 ± 3.7; *P *= 0.005; stimulated percentage expression: control (*n *= 21), 64.7 ± 6.0 and SLE (*n *= 39), 48.6 ± 4.5; *P *= 0.03; Figure 5). Unlike the MR, the DC-SIGN expression did not correlate with FD uptake, either in control DCs or lupus DCs (data not shown). Control DCs and SLE DCs showed a strong correlation between MR and DC-SIGN expression (control, *r *= 0.75 for unstimulated cells and *r *= 0.62 for stimulated cells, *P *< 0.005; SLE, *r *= 0.32, *P *= 0.03). Additionally, only stimulated DCs exhibited a correlation between DC-SIGN expression and the SLE Disease Activity Index score (*r *= -0.35, *P *= 0.04).

### Lupus dendritic cells have normal uptake of zymosan A particles

As zymosan A can be taken up by the MR [26], we determined whether lupus moDCs displayed diminished zymosan uptake. There was no significant difference in zymosan uptake between control individuals and lupus patients, either as determined by the mean fluorescence intensity or by the percentage of fluorescein positivity (percentage positivity: control, 40.6 ± 5.3 and SLE, 42.0 ± 5.15; *P *= 0.85; mean fluorescence intensity: control, 286.2 ± 86.5 and SLE, 295.6 ± 39.4; *P *= 0.90).

## Discussion

A growing body of literature is defining the spectrum of abnormalities associated with DCs in SLE. Lupus DCs exhibit an aberrantly activated and mature phenotype [21,27]. As DC maturation is associated with increased migratory capacity, this phenotype may account for the decreased numbers of circulating DCs detected in the blood of SLE patients [28,29] as well as for the increased numbers found in affected organs [30,31]. DC maturation also results in downregulation of antigen uptake machinery and diminished phagocytic capacity.

Our findings of decreased FD uptake by moDCs from SLE patients are therefore consistent with an overactivated phenotype. This impaired uptake capability, however, is not exclusively a function of maturation status, as FD uptake did not correlate with expression of maturation markers in SLE DCs, whereas it did in control DCs. There thus appears to be a lectin phagocytosis abnormality by lupus DCs that is independent of the maturation status.

FD uptake by moDCs has been shown to occur primarily via the MR, although fluid phase pinocytosis also contributes [32]. We demonstrated that SLE moDCs exhibit decreased expression of MR compared with control DCs. As expected, decreased MR expression correlated with low FD uptake. Additionally, these deficits appear to be associated with active disease activity. Whether low MR expression and associated diminished lectin uptake capacity are pathogenic in active lupus and/or the result of other systemic abnormalities present during disease activity is unclear and warrants further investigation.

We also found downregulation of an additional CTLR, DC-SIGN, indicating a more global defect in expression of members of this family. Interestingly, we detected no decrease in surface expression of CR3 or any of the Fcγ receptors. This is not necessarily surprising; although common variants of these genes alter lupus susceptibility in large population studies [20], specific quantitative or functional receptor deficits associated with these allelic variants have yet to be identified.

A number of exogenous factors of potential relevance in lupus can affect MR expression. In particular, medications used to treat SLE could contribute to the phenotypic differences observed in circulating DCs and monocytes. MoDCs cultured in the presence of dexamethasone exhibit upregulated MR expression, a more globally immature phenotype, and higher endocytic activity [33]. We might therefore expect steroid treatment to result in increased MR expression. No association between steroid use and MR expression could be detected, however, either by analysis of patient steroid use or with *in vitro *treatment of control DCs. Additionally, exposure to other immunosuppressive agents could not account for the downregulation observed in CTLRs.

IFNγ downregulates transcription [34] and surface expression [35] of the MR. As peripheral blood IFNγ levels are elevated in SLE patients and have been shown to correlate with nephritis [25], this could be of potential relevance. Indeed, we did document lower MR expression on DCs from patients with high serum levels of IFNγ. Therefore, while clearly there exists an intrinsic deficit in receptor expression by lupus DCs, IFNγ may contribute to aberrant MR expression in the subset of patients with high serum levels.

The functional consequences of decreased MR expression in SLE DCs are unclear, particularly with regards to susceptibility to infection. *In vitro *transfection studies have found the MR to be sufficient for uptake of various pathogens such as *Candida *sp., *Pneumocystis*, and others [36,37]. Studies with MR knockout mice, however, reveal no evidence of increased predisposition towards infections such as *Pneumocystis *[38], *Candida albicans *[39], and *Leishmania *sp. [40] – although a recent study has found hastened mortality from cryptococcal infections [41]. This may be due to considerable redundancy in receptor-mediated uptake of pathogens, with various other receptors able to perform similar phagocytic functions as the MR. We were unable to demonstrate any significant differences between lupus DCs and control DCs in ability to uptake zymosan, an MR ligand – probably for that reason. Decreased MR expression in combination with the other receptor deficits and immunologic aberrancies seen in SLE, however, could still contribute to the overall increased susceptibility of patients to assorted infections.

MR deficiency results in increased circulating lysosomal hydrolases, which indicates that these molecules may be necessary for certain aspects of glycoprotein homeostasis [11]. Surface glycoprotein rearrangement is an important step in normal cellular apoptosis/necrosis [42]. Dysregulated apoptosis has been strongly correlated with the development and perpetuation of autoimmunity in SLE [43]. Additionally, antibodies against glycoproteins have pathologic relevance in SLE [44]. Aberrant glycoprotein processing could therefore have implications in lupus pathogenesis, and future studies will assess this possibility.

## Conclusion

We have demonstrated that monocyte-derived DCs from patients with SLE have diminished phagocytic capacity associated with decreased expression of specific CTLRs. This is an important addition to our understanding of the many pivotal roles DCs play in lupus immunopathogenesis. Decreased phagocytosis of apoptotic material and other normally harmless self-antigens could result in an autoimmunity-promoting milieu with loss of tolerance, inappropriate autoantigen presentation and, ultimately, the serologic and clinical manifestations characteristic of SLE. Additionally, while individual receptors may not be exclusively responsible for clearance of individual pathogens, aberrant phagocytic machinery and uptake capacity could still contribute to inadequate responses to harmful pathogens.

## Abbreviations

BSA: bovine serum albumin; CR3: type 3 complement receptor; DC: dendritic cell; DC-SIGN: dendritic cell-specific intercellular adhesion molecule-grabbing nonintegrin; ELISA: enzyme-linked immunosorbent assay; Fc: crystallizable fragment; FD: FITC-dextran; FITC: Fluorescein isothiocyanate; IFN: interferon; IL: interleukin; CTLR: C-type lectin receptor; mAb: monoclonal antibody; moDC: monocyte-derived dendritic cell; MR: mannose receptor; PBS: phosphate-buffered saline; SLE: systemic lupus erythematosus; TNF: tumor necrosis factor.

## Competing interests

The authors declare that they have no competing interests.

## Authors' contributions

SUM, KR and ST performed all experiments and analyzed the data. SUM drafted the manuscript. MJK conceived and designed the study and helped to draft the manuscript. All authors read and approved the final document.
